# PREDICTORS OF MORTALITY RISK AMONG MEDICARE ADVANTAGE ENROLLEES

**DOI:** 10.1093/geroni/igz038.2654

**Published:** 2019-11-08

**Authors:** Soyoung Choun, Lan Doan, Diana J Govier, Karen Hooker, Carolyn Mendez-Luck, Shelbie Turner, Veronica L Irvin

**Affiliations:** Oregon State University, Corvallis, Oregon, United States

## Abstract

Overall all-cause mortality rates have declined significantly in past decades among individuals aged 65 and above in every racial and ethnic group. We explored demographic, overall health, and disability development as predictors of mortality in Medicare beneficiaries enrolled in Medicare Advantage plans. We used data from the 2014-2018 Medicare Health Outcomes Survey, a nationally representative panel survey with a two-year follow-up, administered by the Centers for Medicare and Medicaid Services. Our sample consisted of 1,273,494 community-dwelling adults aged 65 and older (Mage = 74.5 years, age range: 65-109 years) enrolled in Medicare Advantage plans. Mortality was assessed over a 2-year follow-up period. We used Cox proportional hazards regression analysis to predict risk of all-cause mortality by demographics, self-rated health, chronic health conditions, smoking status, and activities of daily living (ADLs). Among all participants, the mortality rate was 7.0% (n = 88,058) at 2-year follow-up. Advanced age and being male were significantly associated with greater risk of mortality, while higher levels of education and income were inversely associated with mortality. Controlling for other factors, white adults had higher mortality risk than black or African American, Hispanic, and Asian older adults. Individuals who were unmarried, had lower self-rated health, had more chronic health conditions, smoked, and had more ADL limitations had higher mortality risk. Our findings suggest that sustained health and better functional capacity are important elements in decreasing the risk of mortality in older adults.

